# A structured group intervention (TüTASS) with focus on self-perception and mindfulness for children with autism spectrum disorder, ASD. A pilot study

**DOI:** 10.1007/s00406-021-01281-9

**Published:** 2021-07-08

**Authors:** Laura Drüsedau, Anja Schoba, Annette Conzelmann, Alexander Sokolov, Martin Hautzinger, Tobias J. Renner, Gottfried M. Barth

**Affiliations:** 1 Department of Child and Adolescent Psychiatry, Psychosomatics and Psychotherapy, University Hospital of Psychiatry and Psychotherapy, University of Tübingen, Tübingen, Germany; 2 Department of Psychiatry and Psychotherapy, Medical School and University Hospital, Tübingen, Germany; 3 Department of Psychology, Clinical Psychology and Psychotherapy, Eberhard Karls University Tübingen, Tübingen, Germany

**Keywords:** Autism, Group psychotherapy, Body perception, Mindfulness, Children, Emotion

## Abstract

Autism spectrum disorders (ASD) represent a set of long-lasting severe neurodevelopmental conditions and effective therapeutic interventions are needed. Recent research points to the importance of including mindfulness-based elements to improve emotion and body perception in the psychotherapy of patients with ASD. Therefore, we developed a structured group psychotherapy program *The Tübinger Training for Autism Spectrum Disorders* (*Tübinger Training für Autismus-Spektrum-Störungen*; *TüTASS*) which focuses on mindfulness-based training elements. This pilot study accompanying the TüTASS reports the first results on the feasibility of the program with a pre-post comparison of 25 treated children with ASD. The psychometric assessment comprised five standardized questionnaires/ scales evaluating on the basis of parents and patients self-reports the child’s social responsiveness, behavior, strengths and difficulties, quality of life, and depressive symptoms before and after training completion. The results indicated that upon training completion, symptoms with respect to emotional and social problems, externalizing behavior, and attentional and schizoid-compulsive behavior substantially declined. In a questionnaire assessing feasibility and quality of the group training, patients and parents found the therapy highly beneficial, especially as to the focus on emotions and body, and strengths and failures. This training program was developed to bridge the gap of lacking mindfulness-based interventions with the aim to optimize the course of ASD, especially with respect to behavioral disturbances and social-emotional problems.

## Introduction

The prevalence of children with Autism Spectrum Disorders (ASD) is increasing with a currently reported prevalence rate of 0.6–1.0% [1–5]. Children with ASD show persistent patterns of deficits in social interaction as well as impaired social communication. These can cause severe difficulties with their social interactions in various settings such as school or family life [5]. Core features of ASD also include repetitive and restrictive behaviors, interests or activities [6–8].

Several neuropsychological theories have been proposed to account for the difficulties in social cognition and interaction of individuals with ASD such as an impaired theory of mind (e.g., [9]). Perception of emotions and body perception are central to the theory of mind and its impairment in children with ASD. Indeed, substantial difficulties have been reported in emotion recognition. These include poorer recognition of emotions in facial expressions and body language of other people [10–12] as well as the worse perception of their own emotions [13–15]. Furthermore, empathy seems to be reduced in individuals with ASD [16, 17]. However, Poustka and colleagues [18] showed that adolescents with ASD were impaired in their cognitive empathy but did not differ from controls in their affective capacity for empathy. In addition, difficulties of children with ASD have been reported with regard to emotion regulation [19].

So far, little research has been done on body perception in individuals with ASD. Some studies, however, report atypical sensory and perceptual processing features in children with ASD such as perceptual distortions and hypo- and hyperreactivity to sensory, including interoceptive stimuli [1, 20–24]. These might also be associated with motor difficulties [23]. Motor abnormalities in various areas such as fine motor skills, walking, ball skills such as catching and throwing and motion perception are regularly described in people of the autism spectrum and associated with basal disorders. Thus, basal motor skills are affected, but especially those that include top-down processes such as cognitive control and executive functions.

Therefore, treatment strategies are needed that focus on an improvement of emotion and body perception in children with ASD. When we started developing TüTASS such strategies were missing in specialized handbooks and manuals. At the same time, research shows an increasing number of group interventions for children with ASD. These focused on different deficits of individuals with ASD such as the theory of mind [25] and social skills [26–30]. We did not find any studies that evaluated the effectiveness of body perception and mindfulness exercises in children with autism. There have been only a few interventions for adults working with these concepts. In adults with ASD after participating in a mindfulness-based intervention, findings suggest a reduction of depression, anxiety and stress [31–33]. Also, mindfulness-based interventions have been used successfully in children with a number of mental disorders, other than ASD. They are based on the concept of mindfulness that in this psychotherapeutic respect is defined as conscious experience [34, 35]. These interventions employed similar approaches as those addressing children with anxiety and ADHD [36–40] However, all these interventions missed the specificity of ASD in children.

There are only a few studies about group psychotherapies for children with ASD, reporting mixed results concerning therapy efficacy. Some evidence could be found for the efficacy of social skills groups for children and adolescents with ASD [41]. Then, the results of a first large (*n* = 228) confirmatory, multicenter randomized controlled trial for a short-term (12 sessions) ASD-specific add-on group-based psychotherapy have been reported [42]. This psychotherapy appeared to be efficient in male high-functioning children and adolescents with ASD with regard to their social responsiveness as measured by the parent-rated Social Responsiveness Scale (SRS) [42]. So when we started there was, as to our knowledge, no report on any mindfulness-based interventions intended specifically for children with ASD. In addition, there had been no attempt of developing a group training which addresses difficulties in body perception and emotion regulation in this population. In the meantime, interest in corresponding interventions has increased. However, the implementation of concepts including self-perception and mindfulness specifically in ASD is still a relatively new research area. There is also evidence that mindfulness exercises could be beneficial to patients with ASD (e.g. [7, 43]. However, further research on the efficiency and identification of the potential role of self-perception and mindfulness measures for this specific population is needed [7, 43, 44].

Based on this background, we developed *The Tübingen Training for Autism Spectrum Disorders* (*Tübinger Training für Autismus-Spektrum-Störungen*, TüTASS) for groups of 6 to 8 children with ASD. The aim of the present study was to investigate the feasibility, patients’ and parents’ acceptance, and effectiveness of the TüTASS program. We hypothesised that the training would lead to an improvement of ASD and associated symptoms of the participants.

## Methods

### Participants

Patients were recruited from the Department of Child and Adolescent Psychiatry, Psychosomatics and Psychotherapy, University of Tübingen, Germany. Additionally, local education authorities of Tübingen and Reutlingen, practitioners and local associations were asked to inform potential participants. Participants in the study were required to meet the following inclusion criteria: age 7–12 years and a confirmed primary diagnosis of ASD. Patients who entered the study were re-diagnosed by experts from the Department’s autism outpatient unit according to international diagnostic standards using German versions of the Autism Diagnostic Observation Schedule (ADOS; [45]) and Autism Diagnostic Interview-Revised (ADI-R; [46]). Patients with primary disorders other than ASD were excluded from the study.

Overall, 30 patients were recruited and took part in four groups with 5–8 children per group. Four patients failed to attend all sessions either for personal reasons (*n* = 2) or conduct issues during the group sessions (*n* = 1), feeling to young (*n* = 1). One patient (*n* = 1) did not fill out the questionnaires after the training. These patients were not included in the statistical analyses. None of the participants dropped out due to the failure to attend > 4 sessions without an excuse or due to inpatient treatment.

Therefore, as shown in Table 1, the group program was evaluated with a total of 25 participants (23 male, two female, aged 10.08 ± 1.32 years, median = 10.00, variance = 1.71, age range 7–12 years) with a mean IQ 100.78 ± 12.59 median = 102.00, variance = 158.72, range 76–139 (CFT 20-R [47], CFT 1-R [48], IDS [49] or HAWIK-IV [50]). 44% of the patients had comorbidities (for patient characteristics, see Table 1).

**Table 1:** Overview of the patient characteristics for study participants

Parameters	Values
Diagnosis	24% Mixed disorders of conduct and emotions (F92) 20% Specific developmental disorder of motor function (F82) 16% Other behavioural and emotional disorders with onset usually occurring in childhood and adolescence (F98) 16% Tic disorders (F95) 12% Specific developmental disorders of speech and language (F80) 8% Anxiety disorder (F93) 8% Hyperkinetic disorders (F90) 4% Emotional disorders with onset specific to childhood (F93) 4% Conduct disorder (F91) 4% Specific developmental disorder of scholastic skills (F81) 4% Nonorganic sleep disorders (F51) 4% Reaction to severe stress, and adjustment disorders (F43)
Gender distribution	M = 23 (92%), F = 2 (8%)
Age	Mean = 10.08 SD = 1.32, median = 10.00, variance = 1.74, range 7–12
Intelligence Quotient IQ (*n* = 23)	Mean = 100.78 SD = 12.59, median = 102.00, variance = 158.72, range 76–139

The study was approved by the local Ethics Committee of the Eberhard Karls University and University Hospital of Tübingen and conducted according to the Declaration of Helsinki. Informed written consent was obtained from all parents and/or care providers of the patients; the participation in the study was voluntary, and the data processed anonymously.

### Intervention

The TüTASS program is an ambulant (outpatient) structured group therapy for children with ASD. Mindful perception of body and emotions was practiced in small groups of 5–8 children during 12 sessions in total (one session per week) of 90 min each. The training was designed to be conducted by two group leaders. The conjunction of content (*emotion* and *body perception*) and setting (*group training*) was specifically intended to create different approaches to *mindful* perception. The aim of the program was to help children with ASD to acquire a better understanding of their emotions and body, and body language of others. This could lead to a reduced occurrence of atypical behaviours as well as improvements in social interaction and thereby, better management of the patients’ daily routine.

The overall training program comprised an introduction and four parts with one to three sessions each (see below). The first portion of a session served as a welcoming ritual. Children and therapists told each other about their current emotional state using a cube with different smileys (sad, happy, angry, neutral, cheeky, and anxious). Furthermore, exercises were discussed which had been prepared at homework. In the second part of each session, new topics were introduced as described below. The session was completed with a closing ritual consisting of mutual feedback and a game. Each participant received a folder where to put worksheets and home exercises. Parents were involved in the therapy as far as possible. Two parent-therapist conferences and an additional appointment per family were held during the training. The overall training program was as follows:

#### Introduction (session 1)

The first session served getting to know each other and the therapists.

#### Part 1: Mindful perception of emotions and body (sessions 2–4)

The difficulties concerning the perception of emotions and body in individuals with ASD have been described. In this first part of the training, mindful perception of emotions and body was trained. During session 2, the participants learned to distinguish different smells, tastes and sounds as well as visual and tactile sensations. Additionally, positive and negative emotions which can be connected with sensations were discussed. In session 3, the connection between emotions and physical responses was presented and discussed. First, the participants were asked which emotions they know and these were collected on a flipchart. Afterwards, the participants tried to locate some emotions such as anger or sadness into parts of their body to achieve a better understanding of their physical reactions. Session 4 served to introduce the topic of mindfulness as attention towards one’s own self. To this end, the children participated in a short exercise of mindfulness followed by a lesson of archery to distinguish and train their attention towards an external goal.

#### Part 2: Characterising one´s perception of emotions and body (sessions 5–6)

In the second part, the participants learned to characterise and sort their perceptions of emotions and body with the aim to facilitate both recognition and expression of emotions. In session 5 to achieve this, the relation was elaborated between emotion and situation. Role plays served to outline situations, in which certain emotions would typically occur, such as being proud after having shot a goal. Following this, more situations according to the depicted emotions were collected. In session 6, the participants were taught to describe and present emotions: Each participant presented an emotion to the group by using pantomime and the group had to recognise the emotion correctly. Finally, a board game served as training to describe emotions and their mimic presentation.

#### Part 3: Coping with the perception of emotions and body (sessions 7–9)

The third part aimed at establishing a connection between behavior and the perception of emotions and body. Impaired emotion regulation and dysfunctional behavior are often found in patients with ASD [19]. Therefore, this part served to discuss dysfunctional behavior and to learn functional strategies for coping with emotions. In session 7, the topics of stress and stress relaxation were learned. The participants tried different strategies of relaxation such as mindfulness exercises, a yoga exercise, and a PMR *(Progressive Muscle Relaxation)* exercise. The following session 8 focused on behaviors, which go along with strong emotions such as anger and fear. Each patient had the opportunity to describe own experiences. In addition, different strategies of coping with strong emotions were discussed and rated. The session concluded with the patients presenting functional strategies in a role play. In session 9, another archery task was offered focusing on strength and lack of strength, and weakness in a physical and mental sense was discussed. The participants also told the group about their strengths.

#### Part 4: Perception of oneself and others (sessions 10–12)

The concluding part of the training served recalling and deepening previous skills of self-perception. In addition, the participants were instructed to extend these skills to the perception of other people’s emotions. In session 10, emotion recognition in other people was trained. Pictures of people with different emotions were presented and discussed. Furthermore, the children practised in pairs to produce and recognise emotions. Session 11 served as a playful summary of the emotion topic. The children played in two teams and received credits for answering questions about emotions and their connection to situations, physical reactions, cognition and behavior. In concluding session 12, the participants were encouraged to work out their individual strategies for the regulation of emotions. Furthermore, they trained giving feedback to each other while doing a massage exercise in which the patients pretend to bake a pizza on the partner's back. A game was used as a positive completion of the training.

### Instruments

The psychometric assessment consisted of five standardized questionnaires evaluating on the basis of parents’ reports social reactivity, behavior, strengths and difficulties, quality of life, and depressive symptoms before and after the participation of the child in the training. The Social Reactivity Scale (SRS [51] was used for assessing autistic core features [25]. The *Child Behaviour Checklist* (CBCL [52] and *Strength and Difficulties Questionnaire (*SDQ) [53] assessed atypical behaviors. The Inventory for Assessment of Quality of Life in Children and Adolescents (ILK [54]) evaluated the quality of life. We also used the Depression Inventory for Children and Adolescents (DIKJ) [55] to assess the severity of depressive symptoms. In addition, both patients and parents evaluated the feasibility and quality of the group training using questionnaires that were administered after training completion. These were specifically designed for the TüTASS and served as a feedback and basis for further development of the training. Due to data anonymization, the questionnaires could not be assigned to the individual participants and therefore feedback from a few dropouts was also included.

### Statistical analyses

All statistical analyses were carried out using SPSS for Windows, Version 25 (IBM SPSS Statistics 22; IBM Corporation). Prior to data processing, all data sets were tested for normality of distributions using the Shapiro–Wilk test. Changes in psychometric scores after the group program were assessed either with paired *t*-tests for normally distributed data or otherwise, with the Wilcoxon tests (both two-tailed, i.e., non-directional) as indicated throughout. A *p*-level of 0.05 was chosen as the criterion of significance.

## Results

### Psychometric assessment

Table 2 represents scores for (sub)scales of questionnaires before and after completion of the training along with the statistical data. As can be seen from Table 2, SRS scores for social motivation improved while those for social awareness, social cognition, social communication and restricted interests and repetitive behavior remained stable. CBCL revealed a decrease in external problems, social problems, thought problems, attention problems and rule-breaking as well as aggressive behavior. Internalizing symptoms were not significantly affected as indicated by CBCL and DIKJ. SDQ specified a decrease in emotional symptoms and, marginally, hyperactivity or conduct problems. ILK showed no improvement in quality of life.

**Table 2:** Clinical measurements before (T0) and upon training completion (T1), mean ± standard deviations, and statistics

Questionnaire	T0	T1	Statistics	Pre-post effect sizes
SRS score SRS SocAwa SRS SocCog SRS SocCom SRS SocMot SRS ResInt CBCL CBCL Int CBCL Ext CBCL WD CBCL SC CBCL AD CBCL SP CBCL TP CBCL AP CBCL RBB CBCL AB SDQ SDQ ESS SDQ CPS SDQ HS SDQ PPS SDQ PS ILK P QOL(0–28) ILK C QOL(0–28) DIKJ	82.40 ± 10.35 77.12 ± 9.84 77.60 ± 8.95 85.20 ± 12.57 79.28 ± 11.77 79.52 ± 11.44 71.82 ± 7.85 69.73 ± 6.53 65.55 ± 8.96 70.41 ± 8.15 62.45 ± 10.19 66.95 ± 9.21 73.95 ± 10.04 71.64 ± 11.00 71.91 ± 11.66 62.34 ± 7.61 67.09 ± 11.39 20.32 ± 7.13 5.04 ± 2.59 4.16 ± 2.12 5.88 ± 2.45 5.28 ± 2.21 5.44 ± 1.96 17.40 ± 3.14 20.40 ± 3.20 52.87 ± 11.59	80.28 ± 9.27 74.84 ± 9.66 75.92 ± 8.36 82.64 ± 10.99 75.72 ± 12.32 77.76 ± 10.88 67.36 ± 8.63 66.95 ± 7.57 60.68 ± 9.01 67.68 ± 9.30 60.45 ± 9.16 64.95 ± 8.99 69.45 ± 8.70 67.82 ± 11.62 67.91 ± 9.16 59.05 ± 6.31 62.50 ± 9.28 17.76 ± 6.62 4.12 ± 2.26 3.60 ± 1.83 5.08 ± 2.45 4.92 ± 2.48 5.28 ± 2.11 17.68 ± 3.05 20.28 ± 3.58 54.04 ± 11.96	*t* = 1.67, *p* = .107, *n* = 25 *t* = 1.58, *p* = .127, *n* = 25 *z* = − 1.31, *p* = .197, *n* = 25 *z* = − 1.62, *p* = .109, *n* = 25 *t* = 2.43, *p* = **.023**, *n* = 25 *z* = − .95, *p* = .205, *n* = 25 *t* = 3.40, *p* = **.003**, *n* = 22 *t* = 1.84, *p* = .080, *n* = 22 *t* = 4.29, *p* < **.001**, *n* = 22 *t* = 1.50, *p* = .149, *n* = 22 *z* = − .95, *p* = .367, *n* = 22 *z* = − 1.25, *p* = .218, *n* = 22 *z* = − 2.39, *p* = **.015**, *n* = 22 *z* = − 2.02, *p* = **.042**, *n* = 22 *t* = 2.28, *p* = **.033**, *n* = 22 *t* = 2.38, *p* = **.027**, *n* = 22 *t* = 3.62, *p* = **.002**, *n* = 22 *t* = 2.98, *p* = **.007**, *n* = 25 *t* = 3.57, *p* = **.002**, *n* = 25 *t* = 1.90, *p* = .070, *n* = 25 *t* = 2.02, *p* = .055, *n* = 25 *t* = 0.83, *p* = .417, *n* = 25 *t* = 0.42, *p* = .679, *n* = 25 *t* = 0.50, *p* = .624, *n* = 25 *z* = − .08, *p* = .950, *n* = 25 *t* = 0.51, *p* = .616, *n* = 23	*δ* = *.33* *δ* = *.32* *δ* = *.28* *δ* = *.32* *δ* = *.49* *δ* = *.25* *δ* = *.72* *δ* = *.39* *δ* = *.92* *δ* = *.32* *δ* = *.22* *δ* = *.28* *δ* = *.57* *δ* = *.51* *δ* = *.49* *δ* = *.51* *δ* = *.77* *δ* = *.60* *δ* = *.71* *δ* = *.38* *δ* = *.40* *δ* = *.17* *δ* = *.08* *δ* = *.10* *δ* = *.03* *δ* = *.09*

*p* < .05 is considered significant (in bold)

*SRS* Social Responsiveness Scale with subscales: *SRS SocAwa* Social Awareness, *SRS SocCog* Social Cognition, *SRS SocCom* Social Communication, *SRS SocMot* Social Motivation, *SRS ResInt* Restricted Interests and Repetitive Behavior, *CBCL* Child Behavior Checklist, *CBCL Int* Internal Problems (Subscales: WD, SC, AD, SP, TP, AP), *CBCL Ext* External Problems (Subscales: RBB, AB), *CBCL WD* Subscale Withdrawn/Depressed, *CBCL SC* Somatic Complaints, *CBCL AD* Subscale Anxious/Depressed, *CBCL SP* Social Problems, *CBCL TP* Thought Problems, *CBCL AP* Attention Problems, *CBCL RBB* Rule-Breaking Behavior, *CBCL AB* Aggressive Behavior; *SDQ* Strengths and Difficulties Questionnaire with subscales: *SDQ ESS* Emotional Symptoms Scale, *SDQ CPS* Conduct Problem Scale, *SDQ HS* Hyperactivity Scale, *SDQ PPS* Peer Problem Scale, *SDQ PS* Prosocial Scale; *ILK* Inventory for assessment of quality of life in children and adolescents, *ILK P QOL(0–28)* Parents—Quality of Life, *ILK C QOL(0–28)* Child—Quality of Life; *DIKJ* Depression Inventory for children and adolescents, SRS, SDQ, ILK scores are based on data of 25 participants, CBCL scores on data of 22 participants, and DIKJ scores on data of 23 participants

### Patient and parent evaluation

Overall, the treatment was very positively evaluated by the children and parents. They reported that they were well motivated to take part in the therapy sessions (scale, 1 = *no, not at all* through 5 = *yes, totally*; children: *M* = 4.00, SD = 1.22; parents: *M* = 3.71, SD = 1.26), that the training was helpful (scale, 1 = *not at all helpful* through 5 = *very helpful*; children: *M* = 3.35, SD = 1.27; parents: *M* = 3.72, SD = 0.89), that it was good to have a group therapy setting (scale, 1 = *very bad* through 5 = *very good*; children: *M* = 4.35, SD = 0.86; parents: *M* = 4.67, SD = 0.59), and that the overall concept was very good (scale, 1 = *not at all sensible* through 5 = *very sensible*; parents: *M* = 4.5, SD = 0.51). Both children and parents indicated that the session focusing on emotions and body and the session focusing on strength and weakness were especially useful. Positive comments on the treatment in general were that the children enjoyed it and profited very much, that the topics were close to their daily life and therefore of great relevance, that the treatment enabled the child to have a better insight and coping with respect to feelings and social situations, and that they wished a continuation of the treatment. There were almost no negative comments, with only a few complains about having homework and the weekly rhythm. The high attendance rate of the children confirms the evidence of a good condition during the training and the high motivation for participation.

## Discussion

The aim of this study was to test feasibility, utility, and acceptance of a new structured group psychotherapy program with a unique focus on emotions and body perception developed specifically for children with autism spectrum disorders. Overall findings show that training was helpful and positively evaluated by the patients. It might be particularly helpful for improving social motivation and reducing emotional problems as well as several behavioral problems. The amount of symptom decline is well in line with other studies on group therapies in ASD patients [25]. Especially external problems such as aggressive behavior decrease. Children and parents reported positive improvements in everyday life. The children were very motivated to participate in the therapy sessions and even wanted the training to be continued. Only the homework itself was rated negatively by the children. A very low drop-out rate indicated the good acceptance of this structured treatment program.

In contrast to individual therapies, group therapies are above all beneficial for practicing social skills [56]. Other autism-specific trainings, such as the social skills training SOSTA–FRA and an outdoor adventure program, also had positive effects on the social motivation of the participants [27, 57]. The improved social motivation, rated by the SRS, is therefore possibly due to the group situation, special tasks in which the children had to support each other and act as a team, the joint development of goals and social responsibility. Our results show no significant decrease in the other subscales (social awareness, social cognition, social communication, and restricted interests and repetitive behavior) or in the overall social responsiveness score reported by the SRS. However, descriptively also here, scores declined after treatment and effects might be significant in larger sample sizes. Complementary components of social skills training might also be helpful and included in further developments of our treatment manual.

With respect to CBCL and partially also SDQ, we found a decrease especially in externalizing behavior. Due to the repeated engagement with feelings and mindful perception, the frustration tolerance might have increased. This might have a positive effect on both anti-social behavior as well as on ADHD-relevant behavior. The methods of tension and relaxation might also be helpful for the participants to better deal with their emotions. This might also cause improvements in emotion control addressing aggressive behavior measured by the CBCL. Dealing with emotions and especially dealing with anger is a fundamental training component in TüTASS. The children playfully learn how emotions affect their interactions with others and within themselves. During the training the children's focus is directed to their own strengths, thus creating a better self-confidence. They also experience the connection of emotions with their body, their thoughts, their behavior and different situations. They learn to better understand their emotions and they are given tools to affect their different emotions and deal with affective problems. Participants learn to recognize, express and control emotions in various role plays as well as to interact with their parents, siblings and classmates.

The decrease in the scale of attention problems from the CBCL and hyperactivity from the SDQ might also result from a better understanding of their own body e.g. by practicing techniques of mindfulness exercises. This might lead to more relaxation and less impulsive behavior. Indeed, mindfulness-based training in ADHD are very helpful [58]. Similar findings are shown by Philipsen et al. [59], Külz et al. [60] and Hülle [61]. Additionally, Cachia’s [43] preliminary findings suggest that mindfulness exercises lead to a reduction of stress and result in positive changes in children with ASD as well as in their parents.

Based on the CBCL TüTASS results in more improvement of external behavior than of internalizing behavior. In the SDQ behavior problems with peers and prosocial behavior could not be improved within the short training period. The focus in training was on reducing negative behavior and practicing behavioral alternatives. The next step in further studies would be to specifically improve prosocial behavior.

The reduced decrease in internalizing problems in CBCL and DIKJ might be explained by the fact that the training not directly aimed at improving depressive behavior. Furthermore, the ILK showed no improvement in quality of life. It is possible that the TüTASS training sessions were too rare to have a significant impact on quality of life and depression in the questionnaires. According to Mesibov and Shea [62], interventions for people with autism tend to be difficult to measure long-term goals, for example, life satisfaction or building relationships between people. However, the feedback from children and parents showed that families have seen improvements and relief in different areas of life. It is interesting that children rate their quality of life considerably better than their parents, both before and after training. Comparable results are also evident in other studies [63, 64].

Some limitations of our results have to be noted. Methodological problems need to be taken into concern. This work was developed as a pilot study. This pilot study was used to test and adjust the design of the full-scale experiment. Therefore, the following limitations of our study were accepted and ought to be discussed. The greatest limitation of the study is the low number of participants and the lack of a control group. With a total of only 25 children participating in the study the statistical data analysis was only possible to a limited extent. Furthermore, missing questionnaires led to an additional reduction in test power. Also, observations were based on self-rating reports of the parents and patients themselves and we did not use blind observer ratings nor did we determine interrater reliability of the diagnostic procedures. A further limitation of the pilot study is the unequal gender distribution which might reflect the higher proportion of males in autism. Nevertheless, the overarching goal of this study to prove therapist and group-independent effectiveness and patient acceptance of this group program before conducting larger controlled multicenter studies was reached. The TüTASS represents a theoretically well-founded, currently discussed therapeutic approach that addresses real deficits of children with ASD. With the first application of the TüTASS in four groups the feasibility of the training was demonstrated. Due to the high interest of participants in further group meetings, we decided to extend and improve the TÜTASS basic training on emotions and self-perception with a TüTASS social competence training. For this training, especially everyday problems of children and families were taken into account such as individual problems, problems within the families and at school. Additionally, elements for the parents are extended. To investigate how the children benefit from the training the revised training is currently under evaluation.

## Data Availability

Data is available from the authors upon request.
